# Phytoconstituents of an ethanolic pod extract of *Prosopis cineraria* triggers the inhibition of HMG-CoA reductase and the regression of atherosclerotic plaque in hypercholesterolemic rabbits

**DOI:** 10.1186/s12944-020-1188-z

**Published:** 2020-01-13

**Authors:** Heera Ram, Noopur Jaipal, Jaykaran Charan, Priya Kashyap, Suresh Kumar, Rashmi Tripathi, Bhim Pratap Singh, Chandra Nayaka Siddaiah, Abeer Hashem, Baby Tabassum, Elsayed Fathi Abd_Allah

**Affiliations:** 1 Department of Zoology, Jai Narain Vyas University, Jodhpur, Rajasthan 342001 India; 2 Department of Pharmacology, All India Institute of Medical Sciences, Jodhpur, Rajasthan 342001 India; 3 UniversitySchool of Biotechnology, GGS Indraprastha University, Dwarka, Sector 16C, New Delhi, 110075 India; 4 Department of Bioscience and Biotechnology, Banasthali University, Banasthali, Rajasthan 304022 India; 5 Department of Biotechnology, Mizoram University, Aizawl, Mizoram 796004 India; 6 DOS in Biotechnology, University of Mysore, Manasagangotri, Mysore, India; 7 Botany and Microbiology Department, College of Science, King Saud University, P.O. Box. 2460, Riyadh, 11451 Saudi Arabia; 8 Mycology and Plant Disease Survey Department, Plant Pathology Research Institute, ARC, Giza, 12511 Egypt; 9 Toxicology Laboratory, Department of Zoology, Govt. Raza P.G. College, Rampur, U.P 244901 India; 10 Plant Production Department, College of Food and Agricultural Sciences, King Saud University, P.O. Box. 2460, Riyadh, 11451 Saudi Arabia

**Keywords:** HMG-CoA reductase, Hypercholesterolemia, Lipid profile, Antioxidants, *Prosopis cineraria*, Atherosclerosis

## Abstract

**Background:**

The HMG-CoA reductase is key enzyme of cholesterol biosynthesis which potentially contributes in management of hypercholesterolemia. The present study was designed to assess the inhibitory effect of phytoconstituents of an ethanolic extract of *Prosopis cineraria* pods on HMG – CoA reductase and regression potential of atherosclerotic plaque.

**Methods:**

Healthy, adult male, albino rabbits in which hypercholesterolemia was induced by supplying the high fat diet and a supplement of cholesterol powder with coconut oil (500 mg/5 ml/Day/kg body weight) for 15 days, were used as a disease model. Phytochemical analysis of an ethanolic extract *Prosopis cineraria* pods was conducted using LCMS, GCMS and FTIR analysis. Further, in-vitro, in-vivo and *in-silico* assessments were performed.

**Results:**

The in-vitro assessment of HMG -CoA reductase activity indicated a 67.1 and 97.3% inhibition by the extract and a standard drug (Pravastatin), respectively. Additionally, an *in-silico* evaluation was made using appropriate docking software and results also indicated as significant interactions of the identified compounds with the target enzyme. Treatment of rabbits with the ethanolic extract of *P. cineraria* pod resulted in significant (*P* ≤ 0.001) reductions in total cholesterol, LDL cholesterol, VLDL cholesterol, and triglyceride. Accordingly, reductions were occurred in atherosclerotic plaque, intima and media of aortal wall along with lumen volume of the aorta significantly increased (*P* ≤ 0.001).

**Conclusion:**

It can be illustrating that the ethanolic extract of *Prosopis cineraria* pod contains potent bioactive phytocompounds might be inhibit HMG – CoA reductase and have regression potential of atherosclerotic plaque.

## Introduction

Appropriate diets and dietary supplements have the potential for use in the management of various metabolic syndromes and their complications [1]. The fast food and/or junk food associated with many developed countries are having a drastic impact on the health of youth, as well as many adults with insufficient exercise. These types of food are typically rich in free fatty acids and contain a large amount of fatty substances, all of which promote diabetes, hypercholesterolemia, cancer, and other metabolic syndromes [2]. In fact, three metabolic syndromes, cardiovascular diseases, diabetes, and cancer are the cause of up to 60–70% of mortality, worldwide [3]. Hypercholesterolemia is an independent risk factor that alone or together with the consumption of unhealthy foods can accelerate the development of atherosclerosis and further resulted in atherosclerotic plaque [4]. An increased generation of intracellular free radicals has also been demonstrated to play an important role in chronic inflammatory responses and atherosclerosis.

Several pharmacological agents are available, however, to manage hypercholesterolemia and atherosclerosis. One of the most widely used agents used for the therapeutic treatment of atherosclerosis are HMG-CoA inhibitors, known as statins [1, 5]. The long term use of statins, however, have been shown to be associated with undesirable side effects [6]. For this reason, there is increased interest to identify alternatives to the use of statins that are reliable, effective, and have no or a low-level of adverse effects. In this regard, herbal formulations have been the centre of focus. Studies have demonstrated that some plants and their products exhibit hypolipidemic potential [7].

For example, the desert plant, *Prosopis cineraria,* has been reported by several researchers to possess numerous medicinal properties, such as antidiabetic, hypoglycemic, anticancer, anti-inflammatory, anti-asthmatic, as well as a myriad of other pharmaceutical properties [8, 9]. Studies of this plant have also indicated that it possesses a number of potent bioactive compounds, including polyphenols, alkaloids, tannins, saponins, and flavonoids [10]. Notably, the pod of *Prosopis cineraria* is a key ingredient panchkuta, a local Indian food dish with proposed healthy attributes without side effects. Therefore, a study was conducted to evaluate the inhibitory effect of an ethanolic extract of *Prosopis cineraria* pods on HMG-CoA reductase, and the antioxidant and anti-atherosclerotic potential of the extract. In-vitro, in-vivo*,* and *in-silico* assessments of the extract were conducted.

## Materials and methods

### Pod procurement, authentication and extraction method

Pods of *Prosopis cineraria* (commonly known as Sangari) were obtained from a local herbal shop and authenticated by an expert in botany. The dried pods were ground with a mortar and pestle and a 70% ethanolic extract was obtained by soxhlation. A sticky extract was obtained and stored under desiccated conditions. Atorvastatin, a commonly prescribed statin, was purchased from a local pharmacy and used for comparative purposes (control). All chemicals were of reagent grade and purchased from Loba Chemie.

### LC/MS chemical analysis

LC-MS based metabolomics is a methodology used for characterizing the chemical fingerprint of herbal plant extracts. In our analysis, several mobile phase sequences were analysed to obtain the most comprehensive elucidation of chromatographic peaks [11].

### Gas chromatography with tandem mass spectrometry (GC-MS/MS) analysis

GC-MS analysis of the ethanol pod extract of *Prosopis cineraria* was conducted using a standard protocol. The sample was injected into a gas chromatograph interfaced with a mass spectrometer (GC-MS) [12].

### FTIR analysis

An FTIR Spectrophotometer (Bruker Co., Germany) equipped with a standard detector and a germanium beam splitter, which was interfaced to a computer and analytical software, was utilized for the analysis. The KBr pellet technique was used to obtain a spectrum was in the mid IR region of 400–4000 cm^− 1^. The spectrum was characterized using the attenuated Total Reflectance (ATR) technique [13].

### In-vitro assessment of HMG -CoA reductase inhibition

The inhibitory effect of the plant extract on HMG-CoA activity in vitro was determined using an HMG-CoA reductase assay kit (Sigma-Aldrich), which is based on a spectrometric measurement. The concentration of a standard HMG-CoA reductase stock solution was 0.50–0.70 nM. Different concentrations (5 μg/ml, 2.5 μg/ml,1.25 μg/ml, 0.62 μg/ml, and 0.32 μg/ml) of the ethanolic extract were mixed with a reaction mixture containing NADPH, HMG-CoA substrate, and HMGR. Pravastatin (Sigma Aldrich co.) was used as a positive control and distilled water served as a negative control [12, 14]. Inhibition activity was calculated according to the following equation.

$$ Units/ mgP=\frac{\left(\varDelta {A}_{340}/ mi{n}_{sample}-\varDelta {A}_{340}/ mi{n}_{blank}\right)\times TV}{12.44\times V\times 0.6\times LP} $$

Where**:**

- **12.44** = εmM - the extinction coefficient for NADPH at 340 nm is 6.22 mM–1 cm–1. 12.44 represents the 2 NADPH molecules consumed in the reaction.
- **TV** = Total volume of the reaction in ml (1 ml for cuvettes and 0.2 ml for plates)
- **V** = Volume of enzyme used in the assay (ml)
- **0.6** = Enzyme concentration in mg-protein (0.50–0.70 mg P/ml)
- **LP** = Light path in cm (1 for cuvettes and 0.55 for plates).

### Induction of hypercholesterolemia in rabbits and the experimental design

New Zealand, white, male rabbits, weighing approximately 1.5 ± 0.2 kg, were used as an animal model. They were acclimatized for 10 days in cages under controlled environmental conditions consisting of a 12-h light/dark regime and a temperature of 23 ± 2 °C. Food was supplemented with green leafy, seasonal vegetables and water. Hypercholesterolemia was induced by oral administration of 500 g of cholesterol powder mixed with 5 ml of coconut oil for 15 days along with a high fat diet [15].

Animals were divided into four groups (*n* = 5) and the experimentation period was 60 days.

- Group 1: Control, treated with only distilled water for 60 days.
- Group 2: Hypercholesterolemic diet for 60 days.
- Group 3: Treatment with ethanolic *P. cineraria* pod extract (400 mg/kg/day) for 45 days after induction of hypercholesterolemia for 15 days.
- Group 4: Treatment with atorvastatin (0.25mg/kg) for 45 days after induction of hypercholesterolemia for 15 days.

### Samples collections of in-vivo studies and planimetric study

After completion of the experiment at 60 days, overnight fasting animals were autopsied under mild anaesthesia. Blood samples were collected directly via a cardiac puncture and kept in both EDTA-coated test tubes and normal tubes for biochemical and hematological assessments, respectively. The vital organs (heart, aorta, kidney, and liver) were removed, fixed in formalin. and processed for histopathological examination. Planimetric studies of the aorta wall, lumen volume and atherosclerotic plaque were conducted using a Camera Lucida [15].

### Biochemical analyses

Serum was separated by centrifugation and stored at -20 °C. After thawing, assessments of total cholesterol [16], triglyceride [17], HDL – cholesterol [18], lipid profile [19], glucose [20], and other significant parameters were determined using standard methods.

### Assessment of antioxidant properties

Serum LPO (lipid peroxidation) was determined by measuring thiobarbituric acid reactive substances (TBARS) and expressed as malondialdehyde (MDA) content, following the method of Ohkawa [21]. Catalase and superoxide dismutase activity were assessed using the Aebi and Markland methods, respectively [22].

### Molecular docking

Molecular docking of HMG-CoA reductase (PDB:1DQ9) with Pravastatin (Standard Drug, Pubchem CID: 16759173) and the dominant phytocompounds in the ethanolic extract, i.e. Prosopilosine (Pubchem CID: 45269734), Prosogerin A (Pubchem CID: 44257586), Cinerin C (Pubchem CID: 101485482), β- Sitosterol (Pubchem CID: 222284), and Gallic Acid (Pubchem CID: 370) were conducted using Autodock 4.2. Water molecule and other co-factors were removed from the target molecule, while hydrogen atoms were added. Ligand was also prepared and docked into the active/ binding site of the target molecule [23].

### Statistical analysis

Results of the biochemical assessments, organs weights, and planimetric studies are expressed as a mean ± standard error of the mean (SEM) and statistical differences were determined by ANOVA and post hoc mean separation tests*.*

## Results

### In-vitro HMG-CoA reductase inhibition assay

**Fig. 1:**
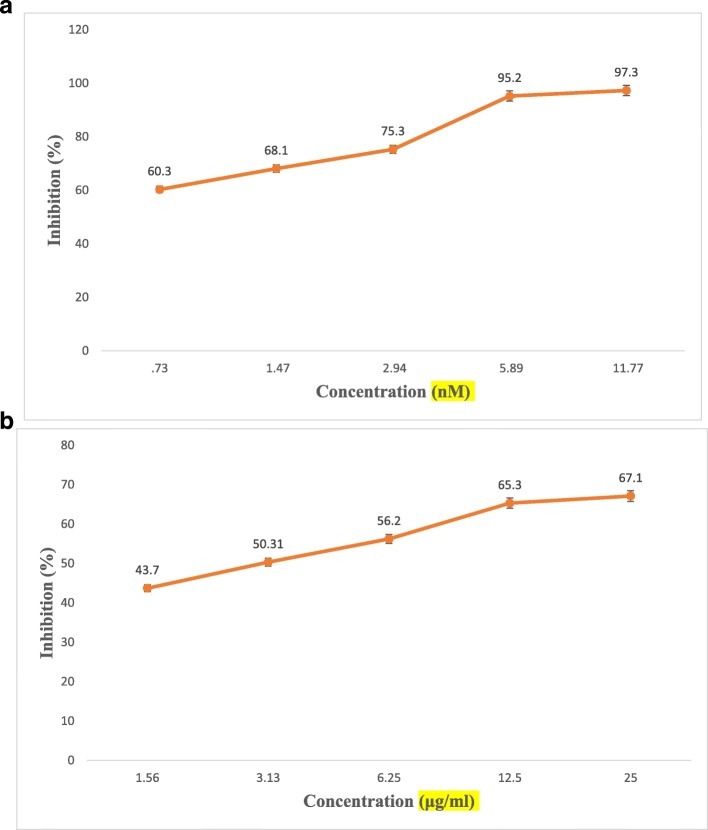
**a** HMG – CoA reductase Inhibition potential of Pravastatin. **b** HMG – CoA reductase Inhibition potential of ethanolic pod extract of *Prosopis cineraria*

The different concentrations of plant extract and the standard inhibitor exhibited a 67.1 and 97.5% level of inhibition of HMG-CoA activity, respectively, as indicated by the product rate per minute, were calculated using the described formula (Figs. 1a and b).

#### LC-MS, GC-MS, and FTIR spectroscopy phytochemical analysis

**Fig. 2:**
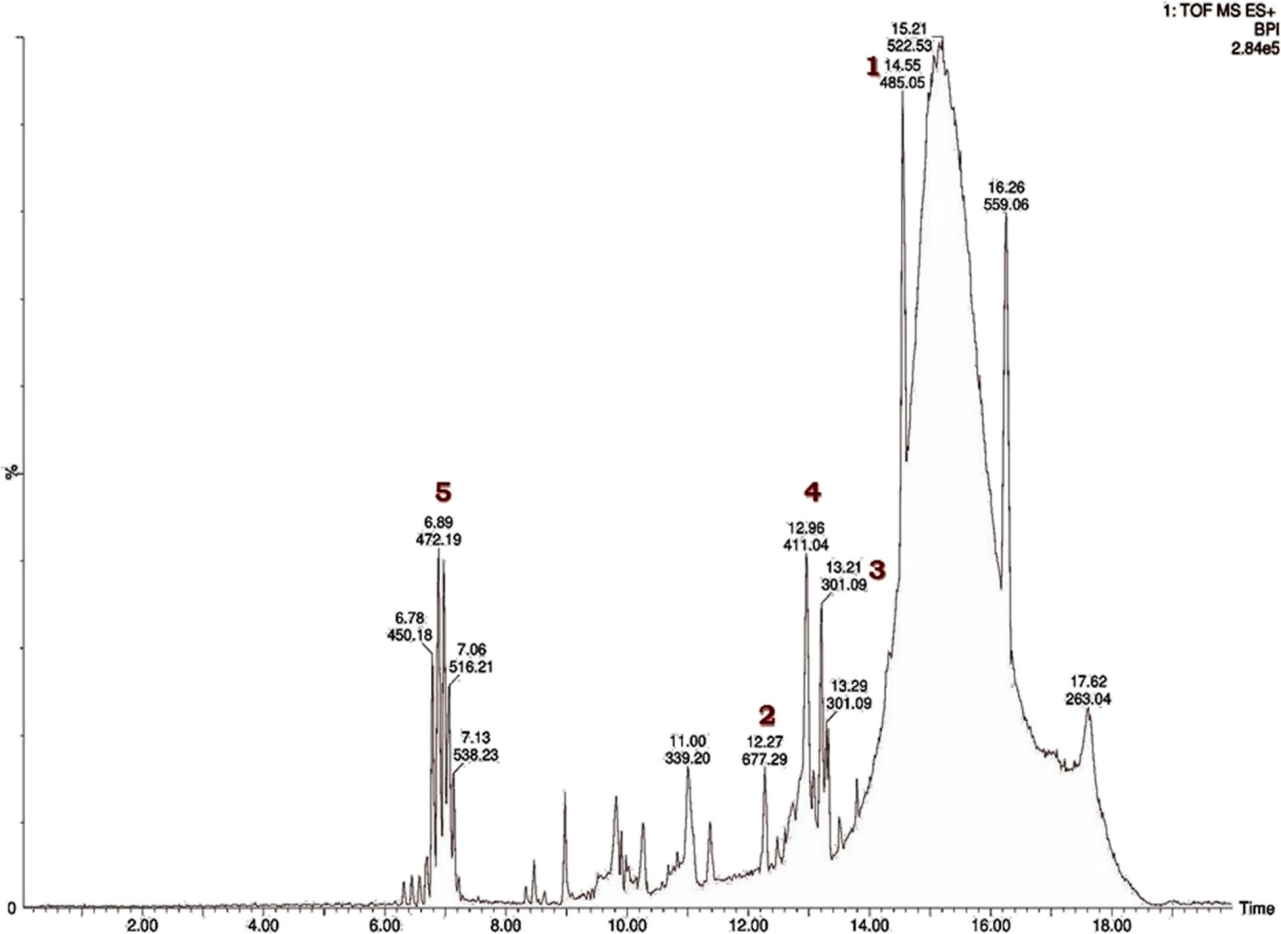
Phytochemical analysis of UPLC chromatogram of ethanolic pod extract of *Prosopis cineraria* of LCMS analysis

**Fig. 3:**
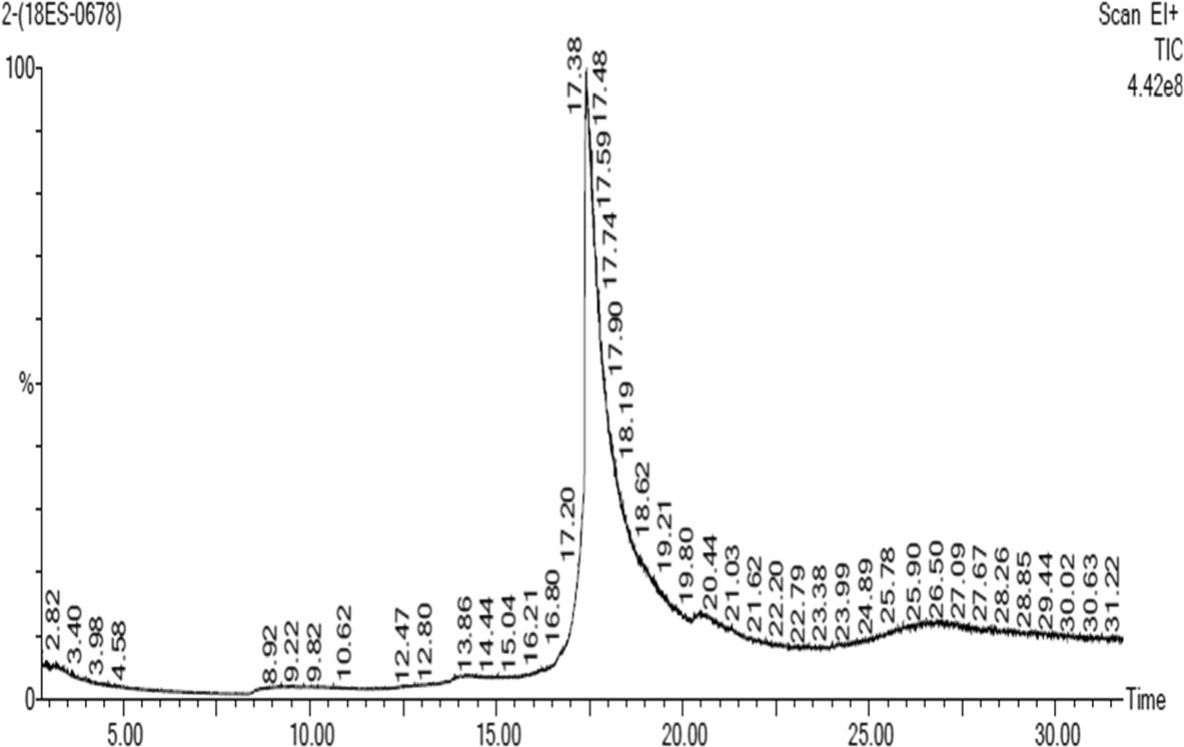
Phytochemical analysis GC/MS of ethanolic pod extract of *Prosopis cineraria*

**Fig. 4:**
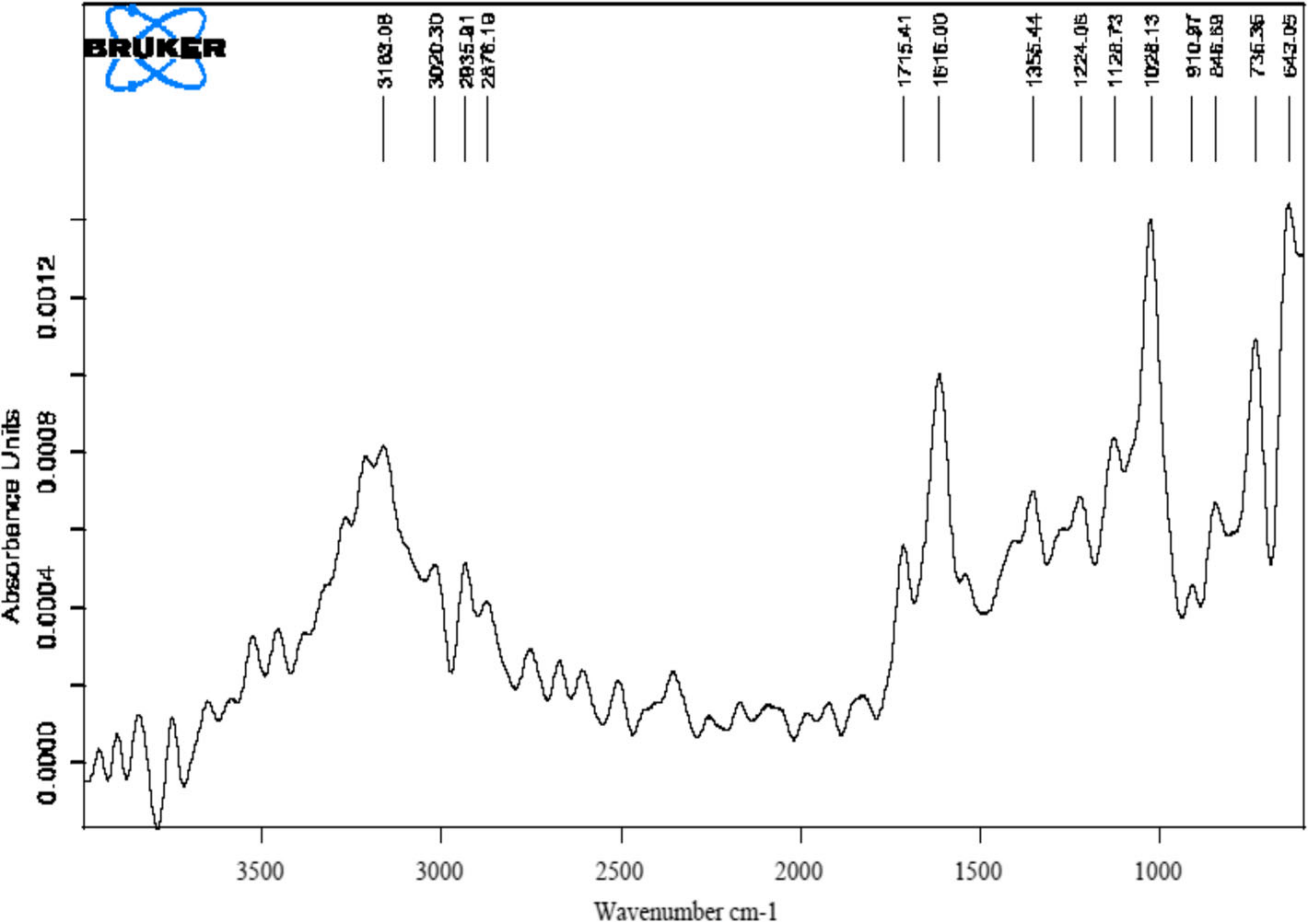
Phytochemical analysis by FTIR of ethanolic pod extract of *Prosopis cineraria*

Using data from the literature and METLIN software, the masses of the constituents were identified based on the monoisotopic mass of the alleged compounds as M and M + H ion using QTOF mass hunter software. The default range for mass documentation was kept above a 100 m/z ratio. The mass spectra exhibited five major peaks in the pod extract (Table 1 and Fig. 2). The GC/MS data also revealed the presence of 3-O-Methyl-D-Glucose (Table 2 & Fig. 3). The functional groups of phytoconstituents in the mid IR region of a 400–4000 cm^−1^wave length were also annotated (Table 3 and Fig. 4).

**Table 1:** Identified phytocompounds of pod extract of *P. cineraria* by UPLC-QTOF analysis

S. No.	Identified compound	Monoisotopic mass	Retention time (min)
1	Cinnerin C	402.168	14.5
2	Prosopilosine	629.586	12.2
3	Prosogerin A	312.063	13.2
4	β- Sitosterol	412.371	12.9
5	Gallic Acid	170.022	6.9

**Table 2:** Identified phytocompounds of pod extract of *P. cineraria* by GC/MS analysis

S. No.	Identified compound	Monoisotopic mass	Retention time (min)
1	3-O-Methyl-D-Glucose	2923	17.419

**Table 3:** FTIR – analysis of ethanolic pod extract of *Prosopis cineraria*

S. No	Wavelength	Functional group
1.	3163.08	CH, NH
2.	3020.30	CH
3.	2935.91	CH
4.	2876.19	= CH3
5.	1715.41	C=O (carbonyl group)
6.	1616.00	C=C
7.	1355.44	Nitro
8.	1224.06	C-O
9.	1128.73	C-O
10.	1028.13	Alkyl halides(R-Cl)
11.	910.97	Alkenes
12.	846.69	Alkyl halides (R-Cl)
13.	735.35	Aromatic compounds
14.	643.05	Alkyl halides

#### Lipid profile

The treatment of rabbits with the ethanolic extract of *Prosopis cineraria* pods resulted in significant reductions in total cholesterol, LDL-cholesterol, triglyceride, VLDL relative to the other treatment groups (Table 4).

**Table 4:** Lipid Profile of ethanolic pod extract of *Prosopis cineraria* treatment

Treatment group	Total cholesterol (mg/dl)	HDL cholesterol (mg/dl)	LDL Cholesterol (mg/dl)	VLDL Cholesterol (mg/dl)	Triglycerides (mg/dl)
Control (Gr.1)	59.5 ± 2.11	21.03 ± 2.9	20.69 ± 1.57	17.78 ± 1.06	69.87 ± 2.05
Hypercholesterolemic (Gr.2)	1216.5 ± 49.3^c^	167.15 ± 8.55^c^	1022 ± 66.41^c^	26.54 ± 8.56^c^	84.7 ± 5.2 ^c^
Ethanolic pod extract of *P. cineraria* (Gr.3)	67.01 ± 4.9 ^c, g^	20.15 ± 2.5 ^c, g^	21.65 ± 2.1 ^c, g^	24.77 ± 1.5 ^c, g^	94.5 ± 11.57 ^c, g^
Atorvastatin (Gr.4)	80.80 ± 4.9 ^c, g^	15.77 ± 1.75 ^c, g^	46.24 ± 7.8 ^c, g^	18.74 ± 4.42 ^c, g^	74.13 ± 2.5 ^c, g^

Data are means ± S.E.M. (*n*=8); a, *P* ≤ 0.05 and c, *P* ≤ 0.001 as compared to the respective control values and g, *P* ≤ 0.001 as compared to the respective values of the Chol diet fed group

#### Effect on antioxidant status

**Fig. 5:**
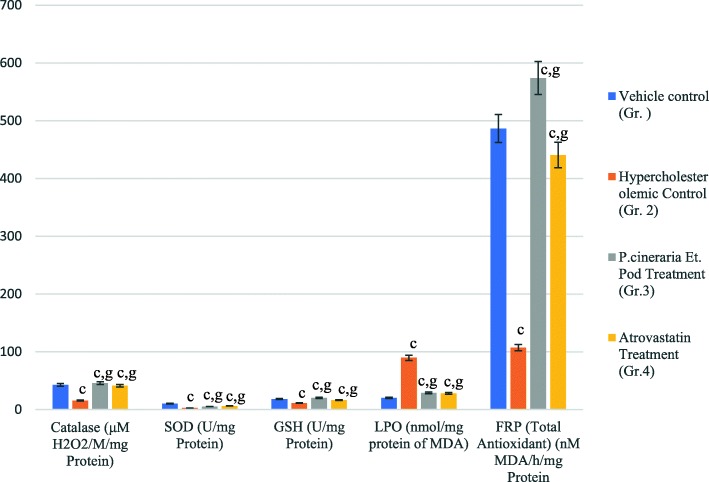
Serum antioxidants levels of ethanolic pod extract of *Prosopis cineraria* in treated groups. (Data are means ± S.E.M. (*n* = b8); a, *P* ≤ 0.05 and c,. *P* ≤ 0.001 as compared to the respective control values and g, *P* ≤ 0.001 as compared to the respective values of the Chol diet fed group)

The MDA content, which is an indicator of lipid peroxidation, was significantly higher (*P* ≤ 0.001) in the hypercholesterolemic group compared to the control group (distilled water). However, a significant reduction in MDA content was observed in the rabbits treated with the ethanolic extract. A significant increase (P ≤ 0.001) in serum SOD and catalase was also observed in the group of hypercholesterolemic rabbits treated with the ethanolic plant extract, relative to the untreated group of hypercholesterolemic rabbits. (Fig. 5).

#### Histopathology

**Fig. 6:**
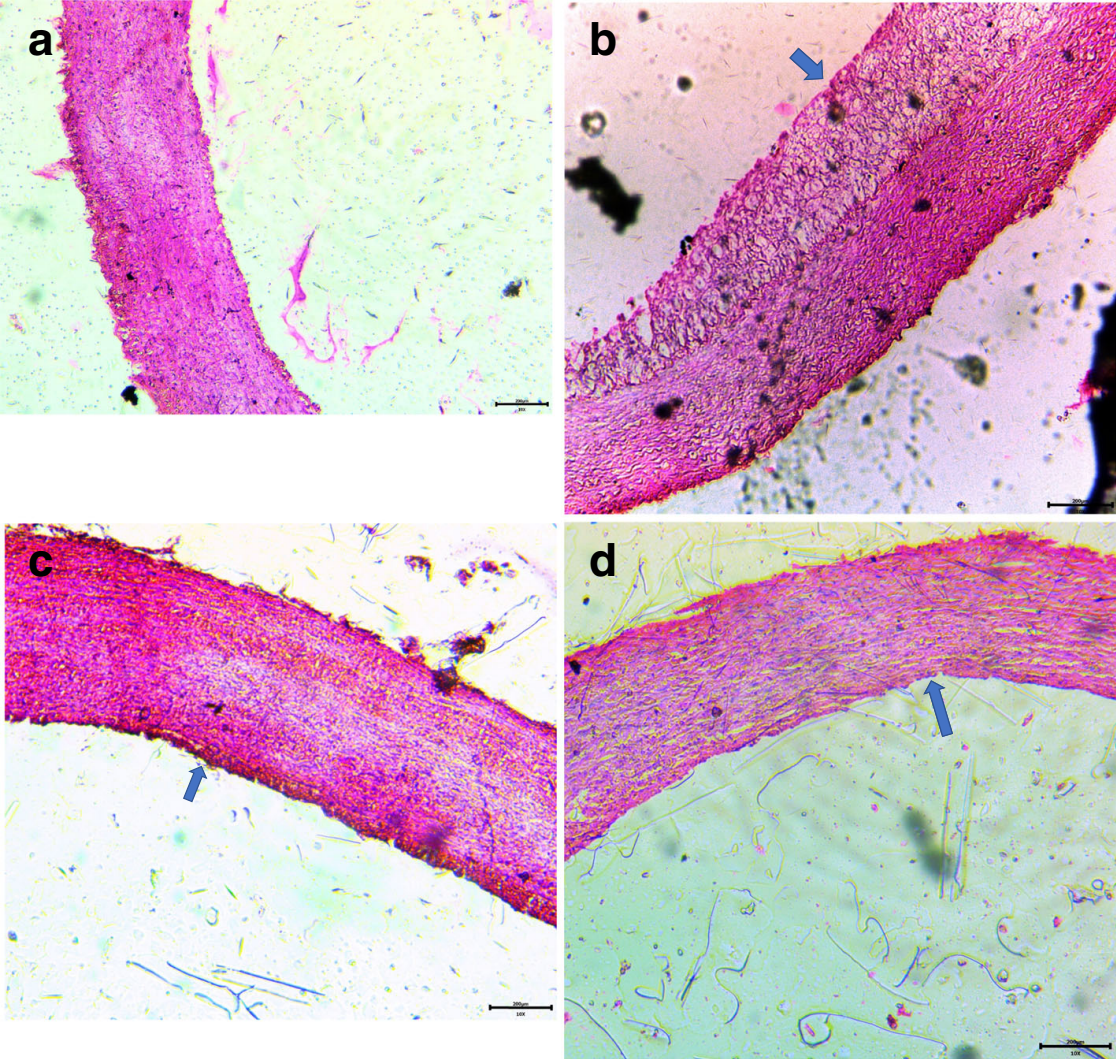
**a** Vehicle control (Gr. 1): Microphotograph of thoracic aorta exhibiting normal histology with aorta wall consists of intima, media and adventitia. (200x H&E). **b** Hypercholesterolemic control (Gr. 2): Microphotograph of thoracic aorta exhibiting atherosclerotic lesions with formation of plaque indicating by arrow, thickened intima contained foam cells and extracellular lipid (200x H&E). **c** Ethanolic *Propopis cineraria* pod extract treatment (Gr. 3): Microphotograph of thoracic aorta depicting regression in plaque indicating by arrow and normalcy in intima and media (200x H&E). **d** Atorvastatin treatment (Gr. 4): Microphotograph of thoracic aorta describing regression in plaque indicating by arrow and normal histoarchitecture of intima and media (200x H&E)

The histological analysis of the aorta revealed three layers, i.e. intima, media, and adventitia, with enlarged lumen volume in the control group (Fig. 6a). In contrast, a bulging structure of atherosclerotic plaque, and reduced thickened media and lumen volume was observed in the aorta of the hypercholesterolemic rabbits (Fig. 6b). Treatment of hypercholesterolemic rabbits with either *Prosopis cineraria* pod extract or atorvastatin resulted in a significant reduction in atherosclerotic plaque and an increase in lumen volume; as well as reconstituted layer thickness of the intima, media, and adventitia layers (Fig. 6c and d).

#### Planimetric study

Fatty plaque with foam cells were seen within the intima region of the aorta of hypercholesterolemic rabbits. In contrast, a significant regression in aortic plaque up to 73.55% along with increased lumen volume up to 23.46% and a somewhat normal histoarchitecture was observed in rabbits treated with the treatment of ethanolic extract of *Prosopis cineraria* pods, compared to the control and standard drug treated groups of rabbits (Table 5).

**Table 5:** Planimetric Dimensions of Ascending Aorta of ethanolic pod Extract of *Prosopis cineraria* Treated Intact Rabbits (Mean of 5 Values ± SEM)

Treatment Groups	Total Wall Area	Lumen	Plaque
Control (Gr.1)	49.42 ± 1.99	50.74 ± 1.44	Nil
Hypercholesterolemic (Gr.2)	78.16 ± 3.61^c^	32.21 ± 2.52^c^	18.59 ± 1.01 ^c^
Ethanolic pod extract of *P. cineraria* (Gr.3)	57.21 ± 5.01 ^c^	44.11 ± 3.23 ^c, g^	4.89 ± 1.38 ^c, g^
Atorvastatin (Gr.4)	53.29 ± 1.01^c, g^	49.31 ± 2.44^c, g^	1.31 ± 0.02 ^c, g^

Data are means ± S.E.M. (*n* = 5); a *P* ≤ 0.05; b *P* ≤ 0.01; c *P* ≤ 0.001; and d nonsignificant as compared to the respective control values. g *P* ≤ 0.001 and h nonsignificant as compared to the respective values of the Chol diet fed group

#### Molecular docking

**Fig. 7:**
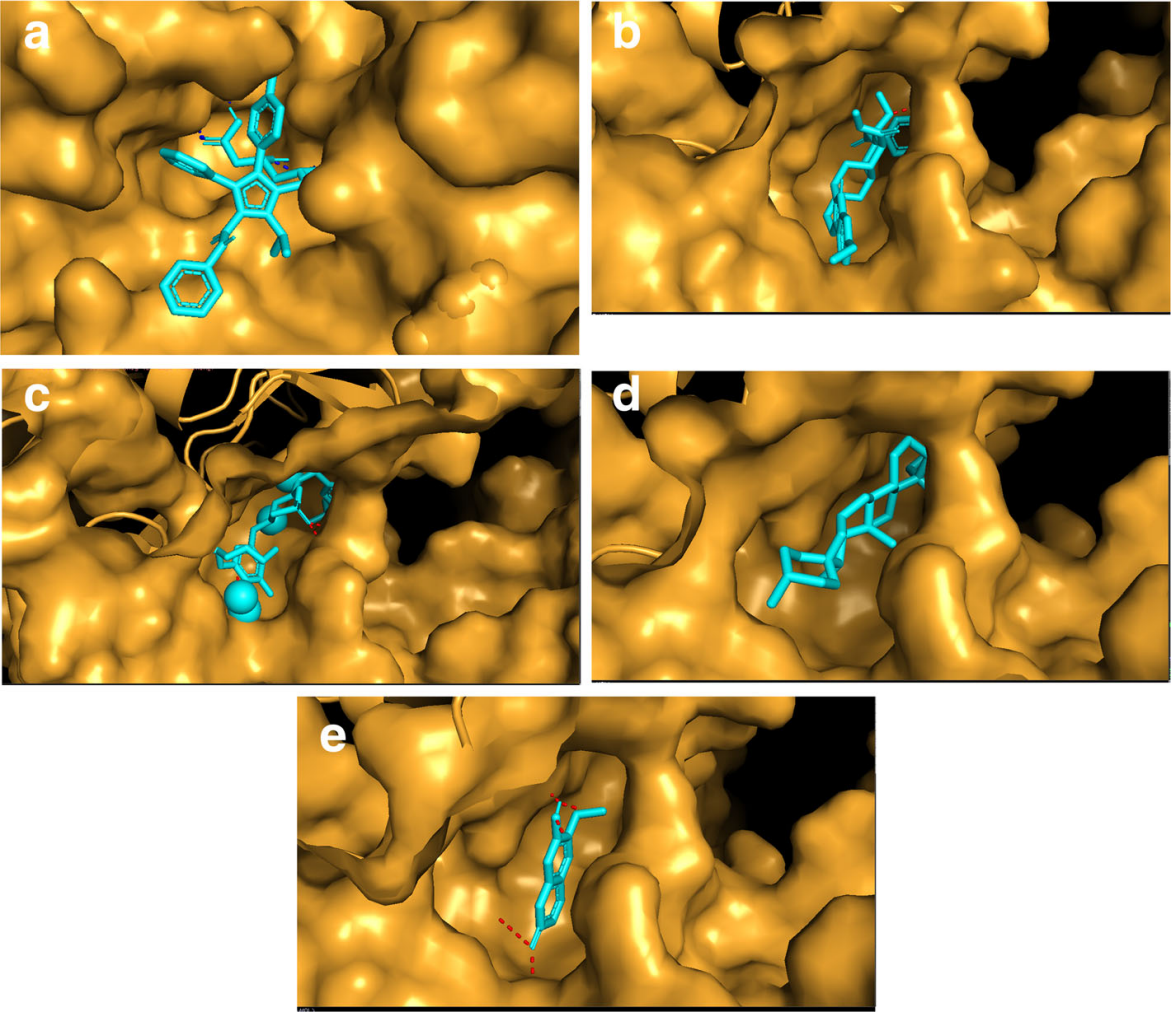
**a** Interaction of Atorvastatin with target enzyme HMG – CoA reductase. **b** Interaction of Pravastatin with target enzyme HMG – CoA reductase. **c** Interaction of Prosogerin - A with target enzyme HMG – CoA reductase. **d** Interaction of Prosopilosine with target enzyme HMG – CoA reductase. **e** Interaction of β-sitosterol with target enzyme HMG – CoA reductase

The molecular docking of HMG-CoA reductase with ligands (standard drug and phytoconstituents of the plant extract) exhibited significant affinity and molecular interactions with the target protein as indicated by the binding energy, number of H-bonds and bond lengths (Table 6 and Fig. 7a-e).

**Table 6:** HMG -CoA reductase molecular docking with standard drugs and phytoconstituents of ethanolic pod extract of *Prosopis cineraria*

S. No.	Ligand	Binding Energy (Kcal/mol)	No. of H-bonds	RMSD
Positive control				
1.	Pravastatin	−7.0	2	1.8
2.	Atorvastatin	−7.5	2	2.4
Phytoconstituents				
3.	Cinnerin C	−6.5	1	1.3
4.	Prosogerin A	−7.9	2	1.4
5.	Gallic Acid	−6.2	2	0
6.	Beta-sitosterol	−6.8	1	1.9

## Discussion

Dietary approaches are frequently used for managing health problems, with specific diets being recommended for the therapeutic treatment of various ailments [24]. Historical treatments are chiefly based on herbal formulations with their efficacy being dependent on the presence of active compounds within the herbal preparation and their bioreactivity [24, 25]. In contrast, improper diet is often the cause of several metabolic disorders and their resulting complications. In modern times, junk food and a fat rich diet have received considerable attention due to their role as a principle factor in hypercholesterolemia and obesity [26]. In the present study, similar symptoms of these atherosclerosis were observed in healthy rabbits after they were subjected to oral administration of cholesterol supplements and a high fat diet. Hypercholesterolemia may result through various mechanisms which results in the progression of atherosclerosis in animal models and higher primates [27–29]. Atherosclerosis is now considered the principle reason for mortality and morbidity worldwide [30]. Numerous therapies presently exist to combat atherosclerosis, such as statins which are HMG-CoA reductase inhibitors, bile acid sequestrants, fibrates, and niacin; all of which are designed to improve the lipid profile [5]. However, these therapeutic agents are often associated with undesirable side effects. In particular, statins are an efficient therapy for treating hypercholesterolemia, through their ability to reduce cholesterol biosynthesis by targeting HMG-Co reductase activity; which is a rate limiting enzyme in the melvonate pathway [31]. The long-term use of statins, however, can result in severe adverse side effects. In contrast, nutraceuticals often exhibit little to no side effects and have been used in the treatment of atherosclerosis, hypercholesterolemia, and various other ailments [32].

In this regard, the desert region of western Rajasthan, India has a native plant, *Prosopis cineraria,* whose pods are used to make an herbal extract which is rich in potent phytochemicals [8, 9]. Bark tissues of *Prosopis cineraria* already have been previously demonstrated to have anti-atherosclerotic activity [8, 33]. In the present study, treatment of hypercholesterolemic rabbits with an ethanolic extract of *Prosopis cineraria* pods resulted in a significant reduction in plaque formation, as well as improvements in their lipid profiles. These results were similar to the those obtained by Ram and co-workers who demonstrated the anti-atherosclerotic activity of *Prosopis cineraria* and *Acacia senegal* bark extracts [15, 33]. It is plausible that these results are due to the presence of potent anti-atherosclerotic and hypocholesterolemic phytoconstituents that reduce lipids and necrotic material, support endothelial repair, or halt the proliferation of vascular smooth muscle cells [34–36]. Several mechanisms may be responsible for this therapeutic activity, such as a reduction in high-density lipoprotein cholesterol (HDL-C), destruction of foam cells and macrophages in lymph nodes, and the restoration of the endothelium by neighbouring cells or circulating progenitors [32, 37]. The alteration in the lipid profile and the regression of atherosclerotic plaque by the plant extract was supported by results obtained in the in-vitro HMG-CoA reductase inhibition assay and *in-silico* evaluation conducted in previous studies [38, 39]. The phytoconstituents in the extract were analysed and identified by LCMS, GCMS, and FTIR; which validated the existence of several phytoconstituents identified functional groups that could potentially interact with target enzyme and serve as competitive inhibitors or analogues of substrates [37, 38, 40].

Additionally, oxidative stress and the production of free radicals have been shown to induce degenerative changes that promote endothelial dysfunction and are thus involved in the pathogenesis of atherosclerosis and are also linked to other metabolic syndromes [41, 42]. In the present study, treatment of rabbits with the ethanolic extract increased antioxidant levels; including SOD and catalase activity. The reduction in oxidative stress and the general increase in antioxidant levels may be attributed to the presence of antioxidants in the plant extract and the ability of the phytoconstituents to generally boost the antioxidant system. Several studies have indicated that oxidation-reduction reactions play a vital role in atherogenesis; however, analysing the role of antioxidants only with respect to their reduction potential may be too narrow of a perspective. Park et al., (2015) reported that the α-asarone constituent in plant extracts of *Perilla frutescens* extracts exhibit a strong antioxidant capacity and avert the oxidation of LDL in vitro and in vivo [43]. Additionally, the α-asarone also triggered the macrophage response to LXR and PPAR-γ agonists, by decreasing SR-B1 and increasing the interaction with ABCA-1 and ABCG-1. Thus, the efficacy of α-asarone could be attributed to both its antioxidant potential and its effect on lipid metabolism [44]. It appears that a balance between oxidative stress and antioxidants is needed in the therapeutics used to treat metabolic syndromes. The antioxidant defense system in cells includes catalase, SOD, and GPx, which help in the degradation of NO [45]. Hypercholesterolemia leads to a decrease in SOD and catalase levels [46]. In the present study, treatment of rabbits with an ethanolic extract of *P. cineraria* pods resulted in a significant elevation in catalase and SOD levels, which may have been induced by the free radical scavenging activity properties of the phytoconstituents present in the plant extract. Our results also indicated that LPO (lipid peroxidation) was reduced in rabbits treated with the extract; which again confirms its role in scavenging reactive oxygen species (ROS). It is possible that these effects may be directly due to the free radical scavenging potential of the phytoconstituents in the ethanolic extract [47]. Collectively, results of the present study demonstrate that the phytoconstituents contained in an ethanolic extract of *Prosopis cineraria* pods inhibits HMG – CoA reductase activity and reduces the level of atherosclerotic plaque in hypercholesterolemic rabbits.

## Conclusion

This present study demonstrated that an ethanolic extract of *Prosopis cineraria* pods can reduce serum cholesterol when fed to hypercholesterolemic rabbits. The extract also reduced the level of atheromatous plague in the aorta. Notably, the extract can improve antioxidant enzyme activity and inhibits HMG-Co reductase activity. Therefore, the use of an ethanolic extract of *Prosopis cineraria* pods should be further investigated as an alternative therapeutic agent to statins and other drugs, but one that does not have adverse side effects for the treatment of cardiovascular diseases.

## Data Availability

All data produced during this study are incorporated in this article.

## Abbreviations

CPCSEA: Committee for the Purpose of Control and Supervision of Experiments on Animals
FRAP: Ferric reducing antioxidant potential
FTIR: Fourier-transform infrared spectroscopy
GCMS: Gas chromatography–mass spectrometry
GSH: Glutathione Sulfhydryl (Reduced Glutathione)
HDL: High density lipoprotein
HMG-CoA Reductase: 3-hydroxy-3-methylglutaryl-coenzyme A reductase
IAEC: Institutional Animal Ethical Committee
LCMS: *Liquid chromatography*–mass spectrometry
LDL: Low density lipoprotein
LPO: Lipid peroxidation
QTOF MS: Quadrupole time-of-flight mass spectrometry
SGOT: Serum glutamic oxaloacetic transaminase
SGPT: Serum glutamic pyruvic transaminase
SOD: *Superoxide dismutase*
TBARS: Thiobarbituric acid reactive substances
VLDL: Very low-density lipoprotein
